# Synthesis of lipophilic 1-deoxygalactonojirimycin derivatives as D-galactosidase inhibitors

**DOI:** 10.3762/bjoc.6.21

**Published:** 2010-03-01

**Authors:** Georg Schitter, Elisabeth Scheucher, Andreas J Steiner, Arnold E Stütz, Martin Thonhofer, Chris A Tarling, Stephen G Withers, Jacqueline Wicki, Katrin Fantur, Eduard Paschke, Don J Mahuran, Brigitte A Rigat, Michael Tropak, Tanja M Wrodnigg

**Affiliations:** 1 Glycogroup, Institute for Organic Chemistry, University of Technology Graz, Stremayrgasse 16, A-8010 Graz, Austria; 2 Chemistry Department, University of British Columbia, 2036 Main Mall, Vancouver, BC, Canada V6T 1Z1; 3 Department of Pediatrics, Medical University of Graz, Auenbruggerplatz 30, A-8010 Graz, Austria; 4 Department of Laboratory Medicine and Pathobiology, Sick Kids Hospital, 555 University Avenue, University of Toronto, Ont., Canada M5G 1X81

**Keywords:** chemical chaperones, 1-deoxy-D-galactonojirimycin, iminosugars, lipophilic galactosidase inhibitor, N-modified iminosugars

## Abstract

*N*-Alkylation at the ring nitrogen of the D-galactosidase inhibitor 1-deoxygalactonojirimycin with a functionalised C_6_ alkyl chain followed by modification with different aromatic substituents provided lipophilic 1-deoxygalactonojirimycin derivatives which exhibit inhibitory properties against β-glycosidases from *E. coli* and *Agrobacterium* sp. as well as green coffee bean α-galactosidase. In preliminary studies, these compounds also showed potential as chemical chaperones for GM1-gangliosidosis related β-galactosidase mutants.

## Introduction

**Figure 1:**
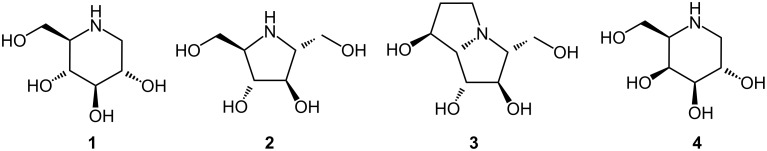
Typical representatives of iminosugars.

Iminosugars such as compounds **1**–**4** (Figure 1) have been shown to be potent glycosidase inhibitors and useful tools for the study of glycoside-hydrolysing enzymes. These sugar mimetics have been found to have anti-viral, anti-cancer, anti-diabetes, anti-infective, as well as insect anti-feedant and plant growth regulatory effects. Because of their diverse properties, iminosugars have enjoyed continuous interest since their discovery in the 1960s. Consequently, many different derivatives have been prepared for biological evaluations via a wide range of synthetic approaches and have been used for various medicinal and biomolecular applications [1–9].

**Figure 2:**
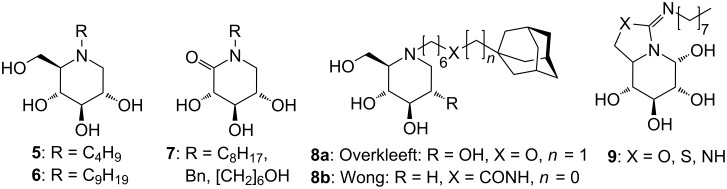
*N*-Modified iminosugars **5**–**9** as potential pharmacological chaperones.

Recently, iminosugars were found to have potential to serve as pharmacological chaperones for the treatment of lysosomal storage diseases in chaperone mediated therapy (CMT) [10]. In contrast to enzyme replacement therapy (ERT), where recombinant enzyme is given to the patient at regular intervals, the iminosugars used for CMT (recently called pharmacological chaperone therapy, PCT) are able to cross the blood brain barrier. This gives the opportunity also to treat types of lysosomal storage diseases involving the central nervous system. Furthermore, CMT is a cost-efficient alternative to ERT. In this context, *N*-alkylated derivatives of 1-deoxynojirimycin [11] such as **5** and **6** (Figure 2) as well as *N*-substituted D-glucono-δ-lactams **7** (Figure 2) [12] have been shown to be highly potent pharmacological chaperones for the potential treatment of Gaucher [13] and Pompe [14] diseases by ‘rescuing’ the related mutant enzymes. Both Wong [15] and Overkleeft [16] have shown that a rather large lipophilic substituent such as the adamantyl group (**8**, Figure 2) attached via an alkyl chain with a chain length from C_3_ up to C_9_ to the ring nitrogen of 1-deoxynojirimycin and isofagomine respectively, can increase the interaction with the lysosomal glycosphingolipid glucocerebrosidase. Interestingly, 5-*N*, 6-*X*-(*N*′-alkyliminomethylene)nojirimycin derivatives where X is O, NH or S such as in structure **9** (Figure 2) also have chaperone activity for Gaucher related mutations [17].

1-Deoxygalactonojirimycin (**4**) was shown to be a candidate for the treatment of Fabry disease, an X-linked inherited lysosomal storage disorder caused by the deficiency of α-galactosidase A activity resulting in the accumulation of globotriaosylceramide, thereby affecting the lysosomes of vascular endothelial cells. Iminosugar **4** can increase α-galactosidase A levels 1.5 to 28 fold in cultured Fabry patient cell lines (baseline α-Gal A levels range from 0–52%) after incubation for five days, as was observed for 49 different missense mutant forms [18–21]. It can also reduce tissue globotriaosylceramide levels in a mouse model [22].

**Figure 3:**
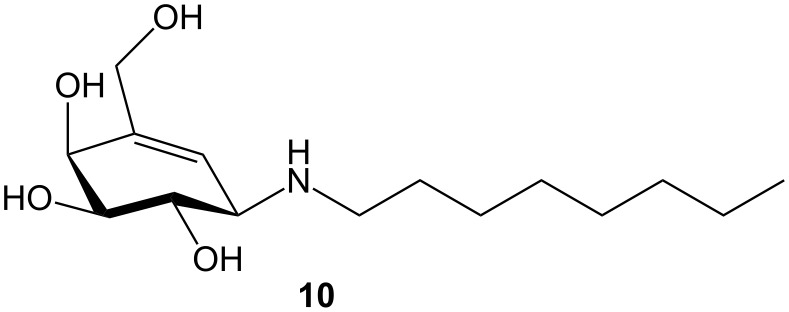
Structure of NOEV **10**.

Suzuki and co-workers found, that *N*-octyl-4-epi-β-valienamine (**10**) (Figure 3), a competitive inhibitor of lysosomal β-galactosidase, when orally administered to GM1-gangliosidosis model mice, is able to enter the brain through the blood-brain barrier and thereby enhancing β-galactosidase activity, reduce substrate storage, and clinically improve neurological deterioration [23].

Our studies revealed that 1-deoxy-D-galactonojirimycin-lysine hybrids, when carrying an aromatic substituent, such as a dansyl moiety, in its nature a lipophilic aromatic substituent, are potent D-galactosidase inhibitors and also show activity with human lysosomal β-galactosidase, exhibiting improvements of the enzyme activity in mutant cell lines [24]. In the course of this work, we became interested in the influence of other lipophilic aromatic substituents on the biological activity of such compounds. Different aromatic acid derivatives were prepared by coupling to the free amine at the terminus of the C_6_ alkyl chain in compound **15**, which is anchored to the ring nitrogen of 1-deoxygalactonojirimycin, to yield derivatives **16**–**19** and **22**. The spacer length of six carbon units has been proven suitable for enzyme recognition in previous studies [25] and was kept constant to compare the different aromatic substituents. Additionally, Wong [15] as well as Suzuki [23] have shown from computational studies, that in case of *N*-substitution on compounds **8b** and **10**, the iminosugar and carbasugar units respectively, were found to interact with the active site of the corresponding enzymes whereas the alkyl chains were located in the distinctly hydrophobic entrance region to the active site. Thus, for comparison, a lipophilic aliphatic *tert*-butyl group in compound **20** was included in this study. In addition to the synthetic approaches, the influence of the lipophilic substituents of the new *N*-modified 1-galactonojirimycin derivatives on their biological interaction with respective glycoside hydrolases are described.

## Results and Discussion

**Scheme 1:**
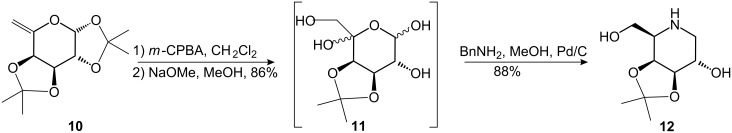
Three-step-synthesis of partially protected 1-deoxy-D-galactonojirimycin derivative **12** from **10** via L-*arabino*-hexos-5-ulose **11**.

The key intermediate for the synthesis of *N*-modified lipophilic 1-deoxygalactonojirimycin derivatives **16**–**20** as well as **22** was the 3,4-*O*-isopropylidene iminosugar **12**. Starting from enol ether **10** [26–27], treatment with *m*-chloroperbenzoic acid gave the 5-*O*-chlorobenzoic ester via the corresponding 5,6-epoxide. This ester underwent hydrolysis under basic conditions to afford the L-*arabino*-hexos-5-ulose **11**, which was immediately used for the next step after brief silica gel purification. The reductive amination and *N*-deprotection of **11** was carried out under an atmosphere of H_2_ with benzylamine in methanol and Pd/C as catalyst to produce 3,4-*O*-isopropylidene-1-deoxy-D-galactonojirimycin (**12**) in an overall yield of 75% (Scheme 1).

**Scheme 2:**
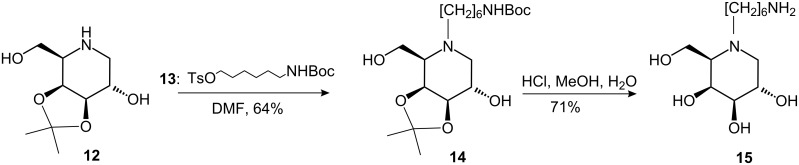
Synthesis of *N*-(6-aminohexyl)-1-deoxygalactonojirimycin (**15**) from **12** via **14**.

Compound **12** underwent *N*-substitution upon treatment with 1-*O*-tosyl-6-*N*-(*tert*-butoxycarbonyl)-6-aminohexanol (**13**) [28] in DMF to give the 1-deoxygalactonojirimycin derivative **14** in 64% yield. The isopropylidene and *tert*-butoxycarbonyl protecting groups were simultaneously removed under standard conditions to afford the desired free amine **15** [25], the key building block for further modifications (Scheme 2).

**Scheme 3:**
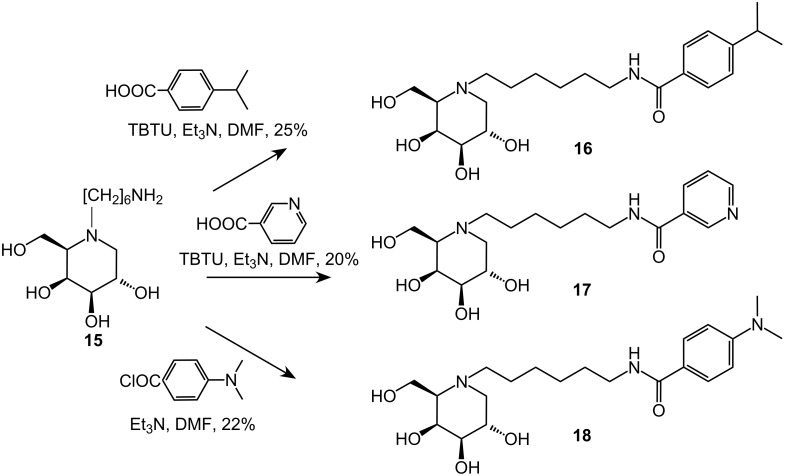
Synthesis of lipophilic 1-deoxy-D-galactonojirimycin derivatives **16**–**18** by chemoselective acylation of **15**.

The chemoselective acylation of the free amine **15** was conducted with three different benzoic acid derivatives in order to investigate the influence of the potential basicity of an additional nitrogen at the aromatic substituent. For the synthesis of compound **16**, 4-isopropylbenzoic acid was reacted with the primary amine under amide coupling conditions with *O*-(benzotriazol-1-yl)-*N*,*N*,*N*′,*N*′-tetramethyluronium tetrafluoro-borate (TBTU) as the coupling reagent in DMF and triethylamine. Likewise, nicotinic acid under the same conditions gave compound **17** in 20% yield. Reaction of **15** with 4-(dimethylamino)benzoyl chloride in DMF and triethylamine afforded derivative **18** in 22% yield (Scheme 3). These unusually low yields for the standard coupling reactions were due to the formation of very polar side products as well as material losses during column chromatography.

**Scheme 4:**
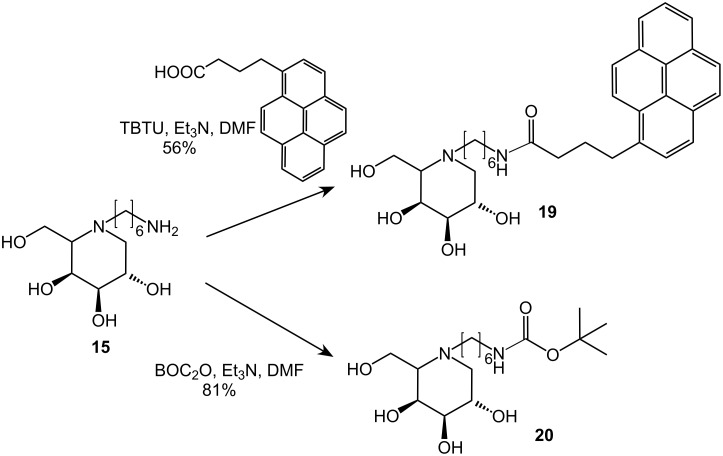
Synthesis of compounds **19** as well as **20** from primary amine **15**.

For increased lipophilicity as well as for analytical purposes, compound **15** was also coupled to 1-pyrenebutyric acid with TBTU in DMF in the presence of triethylamine to give compound **19** in 56% yield. The pyrenyl substituent was chosen because of its potential to serve as diagnostic tool. Conventional BOC-protection of amine **15** gave derivative **20** in 81% yield (Scheme 4).

**Scheme 5:**
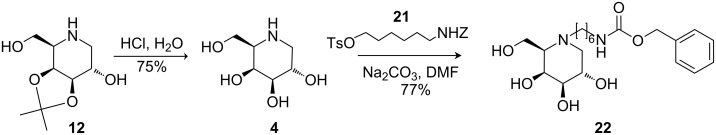
Synthesis of compound **22**.

Aromatic derivative **22** was synthesised from **4** [29–42] (conveniently obtained by deprotection of compound **12** under acidic conditions), in 77% yield by ring nitrogen alkylation with tosylate **21** (Scheme 5) [43].

Inhibition constants of the compounds synthesised are presented in Table 1. The 1-deoxygalactonojirimycin analogues were tested as inhibitors of *Agrobacterium* sp. β-glucosidase/galactosidase and *E. coli* β-galactosidase as well as green coffee bean α-galactosidase (Table 1). All new compounds inhibited the *Agrobacterium* sp. enzyme better than the parent iminosugar **4**. The pyrenyl substituted compound **19** with an extended aromatic system turned out to be the most active inhibitor with a *K*_i_ value of 60 nM against *Agrobacterium* sp. β-glucosidase/galactosidase and 0.25 µM against *E. coli* β-galactosidase. However, the toxicity of this compound clearly requires further evaluation. In general, *N*-substitution does not dramatically affect the inhibitory properties of the derivatives against β-galactosidase from *E. coli*, with *K*_i_ values distributed in the range of the parent iminosugar **4**. No particular trend could be observed in a comparison of compounds **16**–**19** as regards the presence or absence of the additional nitrogen at the aromatic substituent. Iminosugars **16**–**20**, as well as **22**, were less active than the parent compound with α-galactosidase from green coffee beans. However, the *K*_i_ values are still in the low µM range and thus, suitable for use as chemical chaperones. Gratifyingly, compounds **20** and **22** exhibited IC_50_ values of 10.9 µM (*K*_i_ = 2.0 µM) and 3.26 µM (*K*_i_ = 0.72 µM), respectively, with human lysosomal β-galactosidase.

**Table 1:** Inhibitory activities of compounds **16**–**20** and **22** with β-glycosidases from *Agrobacterium* sp. (β-glu/gal Abg), *E. coli* (β-gal *E. coli*) as well as with the α-galactosidase from green coffee beans (α-gal GCB).

Compound	*K*_i_ [µM] β-glu/gal Abg	*K*_i_ [µM] β-gal *E. coli*	*K*_i_ [µM] α-gal GCB

**4**	100	13	0.013^a^
**16**	13	1.3	2.6
**17**	1.5	0.83	2.2
**18**	3.8	1.1	0.49
**19**	0.06	0.25	7.0
**20**	3.0	2.4	4.3
**22**	6.0	0.4	2.2

^a^See reference [7].

In preliminary studies compounds **17** as well as **22** served as chemical chaperone and increased the enzyme activity of a β-galactosidase mutant feline fibroblast cell line up to 5.5 fold when applied at a concentration of 100 µM. Compound **18** was a significantly better chemical chaperone for this mutant increasing the relative enzyme activity 4.8 fold at a concentration of 2 µM.

## Conclusion

We have synthesised new 1-deoxygalactonojirimycin derivatives **16**–**20**, as well as **22**, which feature a C_6_ chain anchored to the ring nitrogen. Different lipophilic aromatic and aliphatic substituents at the *N*-alkyl chain were introduced resulting in an interesting *K*_i_-value profile against β-galactosidases from *Abg* and *E. coli*, respectively, as well as with α-galactosidase from green coffee beans. The *K*_i_ values with human lysosomal β-galactosidase and preliminary data for chaperone activity in a cat fibroblast model of the new compounds suggest that such iminosugar derivatives have interesting potential in the chaperone-mediated therapy of lysosomal storage diseases, such as, for example, GM1 gangliosidosis as well as Morquio B disease, possibly also Fabry’s disease. Further bio-medicinal evaluation and toxicity studies are currently in progress.

## Supporting Information

File 1 Full experimental details and characterisation data
