# Supplementary material for: Synthesis of lipophilic 1-deoxygalactonojirimycin derivatives as D-galactosidase inhibitors
Source: Beilstein J Org Chem. 2010 Mar 1;6:21. doi: 10.3762/bjoc.6.21 (PMC2874416; doi:10.3762/bjoc.6.21)
Supplement: File 1 — Full experimental details and characterisation data [file Beilstein_J_Org_Chem-06-21-s001.pdf]

Supporting Information for

Synthesis of Lipophilic 1-Deoxygalactonojirimycin Derivatives as D-Galactosidase Inhibitors

Georg Schitter[1], Elisabeth Scheucher[1], Andreas J. Steiner[1], Arnold E. Stütz[1], Martin Thonhofer[1], Chris A. Tarling[2], Stephen G. Withers[2], Jacqueline Wicki[2], Katrin Fantur[3], Eduard Paschke[3], Don Mahuran[4], Brigitte Rigat[4], Michael Tropak[4], Tanja M. Wrodnigg[*,1]

Address: Glycogroup, [1]Institute for Organic Chemistry, University of Technology Graz, Stremayrgasse 16, A-8010 Graz, Austria. [2]Chemistry Department, University of British Columbia, 2036 Main Mall, Vancouver, BC, Canada V6T 1Z1. [3]Department of Pediatrics, Medical University of Graz, Auenbruggerplatz 30, A-8010 Graz, Austria. [4]Department of Laboratory Medicine and Pathobiology, Sick Kids Hospital, 555 University Avenue, University of Toronto, Ont., Canada M5G 1X8.

Email: t.wrodnigg@tugraz.at

* Corresponding author.

*

Full experimental details and characterisation data

General

Melting points were determined on a Tottoli apparatus and are uncorrected. Mass spectra were recorded on an Agilent Systems 6120 quadrupole LC-MS with APCI in the positive mode. Optical rotations were measured on a Perkin Elmer 341 with a path length of 10 cm. NMR spectra were recorded at 300.13 MHz (^{1}H), and at 75.4 MHz (^{13}C). $CDCl_3$ was employed for protected compounds and D_2O as well as MeOH-*d4* for free sugars. Chemical shifts are given as delta values employing residual, not deuterated, solvent as the internal standard. The signals of the protecting groups were found in the expected regions and are not listed explicitly. Structures of key intermediates were unambiguously assigned by 1D-TOCSY and HSQC experiments. TLC was performed on precoated aluminum sheets (E. Merck 5554). Compounds were detected by staining with concd H_2SO_4 containing 5% vanillin. TLC of iminoalditols was performed employing a mixture of 10% ammonium molybdate (w/v) in 10% aqueous sulfuric acid containing 0.8% cerium sulfate (w/v). For column chromatography Silica Gel 60 (E. Merck) was used. The free inhibitors were chromatographed in $CHCl_3$/MeOH/H_2O/concd aq ammonia mixtures as indicated.

Kinetic Studies:

The inhibitory activities of new lipophilic 1-deoxygalactonojirimycin dervatives **16** – **20** as well as **22** were tested with three glycosidases. *Agrobacterium* sp. β-

glycosidase was purified and assayed as described [1,2]. Kinetic studies were performed at 37 °C in pH 7.0 sodium phosphate buffer (50 mM) containing 0.1 % bovine serum albumin, using 7.2 x 10^{-5} mg/mL enzyme. Approximate values of K_i were determined using a fixed concentration of substrate, 4-nitrophenyl β-D-glucopyranoside (0.11 mM = 1.5 x K_m) and inhibitor concentrations ranging from 0.2 times to 5 times the K_i value ultimately determined. A horizontal line drawn through $1/V_{max}$ in a Dixon plot of this data (1/V versus [I]) intersects the experimental line at an inhibitor concentration equal to $-K_i$. Full K_i determinations where required, were performed using the same range of inhibitor concentrations while also varying substrate (4-nitrophenyl glucoside) concentrations from approximately 0.015 mM to 0.6 mM. Data were analysed by direct fit to the Michaelis Menten equation describing reaction in the presence of inhibitors using the program GraFit [3].

E. coli β-galactosidase was purchased from Sigma Chemicals. Kinetic studies were performed at 37°C in 100 mM sodium phosphate buffer, pH 7.0 containing 0.1% serum albumin. Rates of reaction were determined as a function of both varying substrate and inhibitor concentration over the concentration ranges indicated below. Data obtained were analysed using a nonlinear regression analysis program GraFit, *p*-nitrogalactopyranoside (K_m = 0.03 mM), 0.045 – 0.3 mM) [4].

Green coffee bean α-galactosidase was purified and assayed as described [5]. Kinetic studies were performed at 37°C in 50 mM sodium phosphate buffer, pH 6.5 containing 0.1% serum albumin. Reactions were initiated by the addition of enzyme (final concentration = 0.3 µg/mL), bringing the total volume to 200µL. Reaction rates were measured by monitoring the release of *p*-nitrophenolate (λ = 400 nm, ε = 7.28 x 10^{-3} M^{-1} cm^{-1}) from PNP-Gal by means of a UNICAM 8700 UV-Vis spectrophotometer equipped with a circulating water bath. Inhibition studies were performed by varying

concentrations of both PNP-Gal and inhibitor. K_i values were calculated by direct fit of the initial rates to the equation below using Grafit [3, 6].

$$\upsilon = \frac{V_{max}[S]}{K_m(1+[I]/K_i) + [S]}$$

3,4-*O*-Isopropylidene-1,5-dideoxy-1,5-imino-D-galactitol (12):

A solution of *m*-CPBA (13.8 g, 61.9 mmol, 77%) in CH_2Cl_2 (150 mL) was dried over Na_2SO_4 and added to a solution of 6-deoxy-α-L-arabino-hex-5-enopyranose (**10**) (10 g, 41.3 mmol) in CH_2Cl_2 (50 mL), and the mixture stirred at room temperature for 4 h. After complete conversion of the starting material (TLC), the reaction was cooled to -18 °C to furnish a white precipitate which was removed by filtration. The filtrate was washed with saturated $NaHCO_3$ solution twice and then with water until the pH of the aqueous phase was neutral. The organic phase was dried over Na_2SO_4, filtered and concentrated to dryness to give crude compound **11** (14.3 g) as a brownish foam. The crude product was dissolved in MeOH (150 mL), cooled to -30 °C and NaOMe (1 M in MeOH) was added dropwise to adjust a pH of ~9-10. After complete conversion the reaction was neutralised by addition of ion exchange resin (Amberlite IR-120 H^+, washed with MeOH). The resin was filtered off, the solution concentrated and purified by silica gel chromatography (CH_2Cl_2/MeOH 16/1, v/v) to give compound **11** (9.8 g, 67 %) which was used for the next step immediately.

Compound **11** (1.6 g, 6.77 mmol) and benzyl amine (875 mg, 8.11 mmol) were dissolved in MeOH (250 ml), Pd/C (200 mg, ~ 0.19 mmol Pd) was added and the reaction mixture was stirred under a hydrogen atmosphere (1 atm) at room temperature for three days. After completion of the reaction (TLC), the catalyst was filtered off and the solution concentrated. Silica gel chromatography

($CHCl_3$/MeOH/NH_3 3:1, 1%, v/v) gave compound **12** (1.04 g, 5.11 mmol, 76 %) as a colourless syrup. $[\alpha]_D^{20}$ = +46.7 (*c* 1.0 in MeOH). δ_H (300 MHz, MeOD): 4.14 (1 H, dd, $J_{4,5}$ = 2.7 Hz, $J_{3,4}$ = 5.3 Hz, H-4), 3.75 (1 H, dd, $J_{2,3}$ = 7.3 Hz, H-3), 3.62 (1 H, dd, $J_{5,6a}$ = 6.0 Hz, $J_{6a,6}$ = 10.9 Hz, H-6a), 3.60-3.50 (1 H, m, H-2), 3.54 (1 H, dd, $J_{5,6b}$ = 7.3 Hz, H-6b), 2.92 (1 H, dd, $J_{1eq,2}$ = 5.1 Hz, $J_{1eq,1ax}$ = 12.5 Hz, H-1eq), 2.89 (ddd, 1H, H-5), 2.25 (1 H, dd, $J_{1ax,2}$ = 10.9 Hz, H-1ax), 1.39 (3 H, s, CCH_3), 1.23 (3 H, s, CCH_3). δ_C (300 MHz, MeOD): 110.5, ($C(CH_3)_2$) 81.5 (C-3), 75.2 (C-4), 71.7 (C-2), 63.5 (C-6), 58.9 (C-5), 49.9 (C-1), 28.5, 26.7 (2 C, C(CH_3)$_2$). *m/z*: 204.24 $(M+H)^+$, $C_9H_{17}NO_4$ requires M + H^+ 204.278.

N-[6-(tert-butoxycarbonylamino)hexyl]-3,4-O-isopropylidene-1,5-dideoxy-1,5-imino-D-galactitol (14):

3,4-*O*-Isopropylidene-1,5-dideoxy-1,5-imino-D-galactitol **12** (220 mg.1.08 mmol) was dissolved in DMF (15 mL), *N*-tert-butyloxycarbonyl-6-aminohexyl-4-toluenesulfonate (**13**) [7] (802 mg, 2.16 mmol) and Na_2CO_3 (424 mg,4 mmol) were added and the suspension was heated to 60 °C for 30 hours. After complete conversion of the starting material, as indicated by TLC, the solids were removed by filtration, the filtrate concentrated under reduced pressure and the resulting slurry subjected to silica gel chromatography ($CHCl_3$/MeOH/NH_3 7:1:1 %, v/v) to give compound **14** (278 mg, 0.77 mmol, 71 %) as a colourless syrup. $[\alpha]_D^{20}$ = + 5.3 (*c* = 0.9 in MeOH). δ_H (300 MHz, MeOD): 4.25 (1 H, dd, $J_{3,4}$ = 4.5 Hz, $J_{4,5}$ = 3.6 Hz, H-4), 3.90 (1 H, dd, $J_{6a,5}$ = 5.2 Hz, $J_{6a,6b}$ = 11.9 Hz, H-6a), 3.85 – 3.79 (3 H, m, H-2, H-3, H-6b), 3.07 – 2.98 (2 H, m, H-6'), 2.93 (1 H, dd, $J_{1eq,2}$ = 3.8 Hz, $J_{1eq,1ax}$ = 11.8 Hz, H-1eq), 2.76 – 2.64 (1 H, m, H-1'), 2.64 – 2.56 (1 H, m, H-5), 2.56 – 2.43 (1 H, m, H-1'), 2.05 (1 H, dd, $J_{1ax,2}$ = 10.4

Hz, H-1ax), 1.46 (3 H, s, C(CH_3)$_2$), 1.44 – 1.34 (8 H, m, H-2', H-3', H-4', H-5'), 1.30 (3 H, s, C(CH_3)$_2$. δ_C (300 MHz, MeOD): 156.1 (C=O), 109.8, (*C*(CH$_3$)$_2$), 80.6 (C-3), 77.3 (C-4), 70.4 (C-2), 61.5 (C-5), 60.6 (C-6), 54.2 (C-6'), 53.4 (C-1), 52.5 (C-1'), 28.4 (2 C, C(*C*H$_3$)$_2$), 28.3, 26.9, 26.5, 26.3 (4 C, C-2', C-3', C-4', C-5'). *m/z*: 403.38 $(M+H)^+$, $C_{20}H_{38}N_2O_6$ requires M + H^+ 403.600.

_N_-(Aminohexyl)-1-deoxy-D-galactonojirimycin (15) [8]:

Compound **14** (3.24 mg, 0.78 mmol) was dissolved in a mixture of MeOH and H_2O (30 ml, 1/1 v/v) and 1 ml of concentrated hydrochloric acid was added to adjust the pH to ~1. If necessary, additional HCl was added. The reaction was stirred at 50 °C for 14 hours. After TLC analysis indicated complete conversion of the starting material, the reaction mixture was evaporated to dryness, the residue taken up in MeOH and evaporated again three times to remove excess of HCl. Silica gel chromatography ($CHCl_3$/MeOH/NH_3 1:1:1/4 v/v) gave compound **15** (139.2 mg, 0.53 mmol, 68 %) as a pale-brown, highly hygroscopic foam after lyophilisation from H_2O. $[\alpha]_D^{20}$ = + 6.5 (*c* 1.5 in MeOH). For NMR data see [7].

_N_-[6-(4-Isopropylbenzoylamino)hexyl]-1,5-dideoxy-1,5-imino-D-galactitol (16):

To a solution of 4-isopropylbenzoic acid (93.9 mg, 0.6 mmol) in DMF (5 mL) containing Et_3N (115.6 mg, 1.14 mmol), TBTU (201.9 mg, 0.63 mmol) was added and the reaction mixture stirred for ten minutes. Consecutively, amine **15** (150 mg, 0.6 mmol) was added and the solution stirred at room temperature for one hour. After TLC showed complete conversion of the amine, the solvent was removed

under reduced pressure and the semi-solid residue subjected to silica gel chromatography ($CHCl_3$/MeOH/NH_3 3/1/1% v/v) to yield compound **16** (62.6 mg, 0.15 mmol, 51 %) as a syrup. After lyophilisation from H_2O, (47.1 mg, 0.12 mmol, 20.2 %) a hygroscopic white foam was obtained. $[\alpha]_D^{20}$ = +5.9 (*c* 2.4 in MeOH). δ_H (300 MHz, MeOD/$CDCl_3$ 2/1 v/v): 7.76 (2 H, d, *J* = 8 Hz, Ph), 7.23 (1 H, d, *J* = 8 Hz, Ph), 3.99 (1 H, dd, $J_{3,4}$ = 2.3 Hz, $J_{4,5}$ = 1.8 Hz, H-4), 3.87 – 3.80 (3 H, m, $J_{2,3}$ = 9.3 Hz, $J_{2,1eq}$ = 4.2 Hz, H-2 H-6a, H-6b), 3.33 – 3.26 (3 H, m, H-3, H-6'), 3.12 (1 H, dd, H-1eq), 3.05 – 2.81 (4 H, m, H-5, H-1', H-1', C*H*(CH_3)$_2$), 2.55 (1 H, dd, $J_{1eq,1ax}$ = 11.2 Hz, H-1ax), 1.66 – 1.48 (4 H, m, H-2', H-5'), 1.42 – 1.25 (4 H, m, H-3', H-4'), 1.18 (3 H, s, CH(C*H*$_3$)$_2$), 1.15 (3 H, s, CH(C*H*$_3$)$_2$). δ_C (75 MHz, MeOD/$CDCl_3$ 2/1 v/v): 168.4 (NCO), 152.1, 131.1, 129.1, 126.6, 125,7 (6 C, Ph), 72.6 (C-3), 69.8 (C-4), 64.4 (C-2), 64.2 (C-5), 59.5 (C-6), 53.2 (C-1), 52.9 (C-1'), 38.8 (C-6'), 33.4 (*C*H(CH_3)$_2$), 28.3, 25.5, 25.4, 21.4 (4 C, C-2',C-3' C-4', C-5'), 22.6 (2 C, CH(*C*H$_3$)$_2$). *m/z*: 409.42 $(M+H)^+$, $C_{22}H_{36}N_2O_5$ requires M + H^+ 409.603.

N-[6-Nicotinoylaminohexyl]-1,5-dideoxy-1,5-imino-D-galactitol (17):

To a solution of pyridine-3-carboxylic acid (59.1 mg, 0.48 mmol) in DMF (15 mL) containing Et_3N (132.4 mg, 1.31 mmol), TBTU (154.1 mg, 0.48 mmol) was added and the solution was stirred at room temperature for ten minutes. Amine **15** (114.5 mg, 0.44 mmol) was added and the mixture stirred for 90 minutes until complete conversion of the starting material was observed by TLC. The solvent was removed under reduced pressure and the residue purified by silica gel chromatography ($CHCl_3$/MeOH/NH_3 3/1/1% v/v) to yield compound **17** (34.3 mg, 0.1 mmol, 21.2 %). After lyophilisation, compound **17** was obtained as a hygroscopic, white foam. $[\alpha]_D^{20}$

= -10.1 (c 1.0 in MeOH). δ_H (300 MHz, MeOD): 8.99 (1 H, dd, J = 0.7 Hz, J = 2.2 Hz, Py), 8.71 (1 H, dd, J =1.62 Hz, J = 4.9 Hz, Py), 8.26 (1 H, dt, J = 1.9 Hz, J = 4.9 Hz, Py), 7.57 (1 H, dd, J = 8.0 Hz, J = 4.9 Hz, Py), 4.01 (1 H, dd, $J_{4,5}$ = 1.8 Hz, $J_{3,4}$ = 3.3 Hz, H-4), 3.88 – 3.79 (3 H, m, H-2, H-6a, H-6b), 3.42 (2 H, t, H-6'), 3.24 (1 H, dd, $J_{2,3}$ = 9.25 Hz, H-3), 3.01 (1 H, dd, $J_{1eq,1ax}$ = 11.2 Hz, $J_{1eq,2}$ = 4.8 Hz, H-1eq), 2.85 – 2.68 (1 H, m, H-1'), 2.58 – 2.47 (1 H, m, H-1'), 2.39 (1 H, ddd, $J_{5,6a}$ = 2.4 Hz, $J_{5,6b}$ = 5.2 Hz, H-5), 2.13 (1 H, dd, $J_{1ax,2}$ = 10.7 Hz, H-1ax), 1.73 – 1.61 (2 H, m, H-5'), 161 – 147 (2 H, m, H-2'), 147 – 130 (4 H, m, H-3', H-4'). δ_C (75 MHz, MeOD): 167.8 (NCO), 152.6, 149.3, 137.2, 132.5, 125.4 (5 C, Py), 77.6 (C-3) 72.6 (C-4), 69.3 (C-2), 65.5 (C-5), 62.7 (C-6), 58.4 (C-1), 54.3 (C-1'), 41.3 (C-6'), 30.6 (C-5'), 28.6, 28.2 (2 C, C-3', C-4'), 25.3 (C-2'). *m/z*: 368.32 $(M+H)^+$, $C_{18}H_{29}N_3O_5$ requires M + H^+ 368.507.

***N*-[6-(4-Dimethylaminobenzoylamino)hexyl]-1,5-dideoxy-1,5-imino-D-galactitol (18):**

A solution of amine **15** (57.6 mg, 0.22 mmol) in DMF (4 mL) and Et_3N (44.7 mg, 0.44 mmol) was cooled to 0 °C and 4-dimethylaminobenzoic acid chloride hydrochloride (53.4 mg, 0.2 mmol) in 1 ml of dry DMF was added dropwise with a syringe. The reaction mixture was allowed to reach room temperature and stirring was continued for two hours. After complete consumption of the acid chloride, as indicated by TLC, 1 mL of MeOH was added and the reaction mixture stirred for 20 minutes. The solvents were removed under reduced pressure and the residue subjected to silica gel chromatography ($CHCl_3$/MeOH/NH_3 3/1/1% v/v). Subsequent lyophilisation from H_2O gave compound **18** (20.8 mg, 0.5 mmol, 23.1 %) as an off-white, highly hygroscopic material. $[\alpha]_D^{20}$ = +5.6 (c 1.6 in MeOH). δ_H (300 MHz, MeOD/$CDCl_3$ 2/1

v/v): 7.62 (2 H, d, J = 8.2 Hz, Ph), 6.79 (2 H, d, J = 8.2 Hz, Ph), 4.01 – 3.95 (1 H, m, H-4), 3.78 (1 H, dd, $J_{2,3}$ = 10.6 Hz, $J_{2,1eq}$ = 4.5 Hz, H-2), 3.75 – 3.66 (2 H, m, H-6a, H-6b), 3.32 – 3.25 (3 H, m, H-3, H-6'), 2.93 - 2.86 (7 H, m, H-1eq, N(CH_3)$_2$), 2.62 – 2.49 (1 H, m, H-1'), 2.46 – 2.31 (2 H, m, H-5, H-1'), 2-10 (1 H, dd, $J_{1eq,1ax}$ = 11.0 Hz, H-1ax), 1.57 – 1.45 (2 H, m, H-5'), 1.44 – 1.34 (2 H, m, H-2'), 1.33 – 1.14 (4 H, m, H-3', H-4'). δ_C (75 MHz, MeOD/$CDCl_3$ 2/1 v/v): 171.3 (N*C*O), 154.7, 129.8, 122.3, 113.6 (6 C, Ph), 76.4 (C-3), 71.4 (C-4), 68.3 (C-2), 63.9 (C-5), 61.7 (C-6), 57.2 (C-1), 53.6 (C-1'), 41.0 (2 C, N(*C*H_3)$_2$), 40.9 (C-6), 29.9 (C-5'), 27.8, 27.3 (C-3', C-4'), 23.9 (C-2'). *m/z*: 410.37 $(M+H)^+$, $C_{21}H_{35}N_3O_5$ requires M + H^+ 410.589

1,5-Dideoxy-1,5-imino-D-galactitol (4):

3,4-*O*-Isopropylidene-1,5-dideoxy-1,5-imino-D-galactitol (**12**) (0.62 g, 3.05 mmol) was dissolved in a mixture of H_2O and MeOH (12 mL, 10/2 v/v), concentrated HCl (2 ml) was added (pH~1) and the mixture stirred at room temperature for 20 hours. The reaction mixture was concentrated and co-evaporated with MeOH. The resulting material was purified by silica gel chromatography ($CHCl_3$/MeOH/NH_3 3:1, 1%, v/v) to give compound **4** (196.5 mg, 1.2 mmol. 53 %) as a colourless syrup.

***N*-[(4-Pyrenebutanoylamino)hexyl]-1,5-dideoxy-1,5-imino-D-galactitol (19):**

To a solution of 1-pyrenebutyric acid (123.4 mg, 0.42 mmol) in DMF (15 mL) containing Et_3N (61.9 mg, 0.61 mmol) and HBTU (144 mg, 0.44 mmol), *N*-(6-aminohexyl)-1,5-dideoxy-1,5-imino-D-galactitol (**15**) (111.9 mg, 0.42 mmol) was added in one portion and the mixture stirred at room temperature for 45 minutes.

When TLC indicated complete conversion of the starting material MeOH (10 mL) was added and stirring continued for 10 minutes. The solvents were removed under reduced pressure and the remaining material subjected to column chromatography ($CHCl_3$/MeOH/NH_3 9/1/1 % - 6/1/1 %). The resulting product was treated with basic ion exchange resin (Merck III, washed with water) to give compound **19** (134.0 mg, 0.3 mmol, 59 %) as white crystals. $[\alpha]_D^{20}$ = +3.6 (*c* 1.1 in MeOH/$CHCl_3$ 1/1 v/v). m.p. = 189 – 191 °C. δ_H (500 MHz, D_2O): 7.19 – 6.60 (9 H, m, pyrenyl), 3.96 – 3.88 (1 H, m, H-4), 3.86 – 3.79 (1 H, m, H-2), 3.59 – 3.47 (2 H, m, H-6a, H-6b), 3.25 – 3.18 (1 H, m, H-3), 3.14 – 3.06 (1 H, m, H-1eq), 2.80 – 2.66; 2.63 – 2.45; 2.42 – 2.26 (8 H, m, H-1ax, H-5, H1', H6', H-10`), 1.78 – 1.62 (2 H, m, H-8'), 1.46 – 1.32; 1.15 – 0.97; 0.83 – 0.5 (10 H, m, H-2', H-3', H-4', H-5', H-9'). δ_C (75 MHz, D_2O): 174.5 (NCO), 135.5, 130.6, 130.2, 129.0, 127.8 126.7, 125.8, 125.2, 124.0, 122.7 (pyrenyl), 72.7 (C-3), 70.0 (C-4), 64.5, 64.1 (2 C, C-2, C-5), 59.3 (C-6), 53.4, 53.1(C-1, C-1'), 39.2 (C-6'), 35.6 (C-2'), 32.1 (C-4'), 28.5, 27.3, 25.7, 25.5, 21.7 (5 C, C-3', C-5', C-8', C-9', C-10'). *m/z*: 533.55 $(M+H)^+$, $C_{32}H_{40}N_2O_5$ requires M + H^+ 533.762

***N*-[6-(*tert*-butyloxycarbonylamino)hexyl]-1,5-dideoxy-1,5-imino-D-galactitol (20):** To a solution of iminosugar **15** (80 mg, 0.30 mmol) in DMF (15 ml) containing Et_3N (121 mg, 1.20 mmol), a solution of BOC_2O (80 mg, 0.37 mmol) was added and the mixture stirred at room temperature for 30 minutes. After TLC indicated complete consumption of amine **15**, MeOH (1 ml) was added and the reaction mixture concentrated under reduced pressure to yield compound **20** (94.9 mg, 0.3 mmol, 86 %) after silica gel chromatography ($CHCl_3$/MeOH/NH_3 3:1:1 %, v/v). $[\alpha]_D^{20}$ = -14.3, (*c* 1.1 in MeOH). δ_H (500 MHz, D_2O): 3.89 – 3.85 (1 H, m, H-4), 3.73 – 3.64 (3 H, m, H-

2, H-6a, H-6b), 3.09 (1 H, dd, $J_{2,3}$ = 9.1 Hz, $J_{3,4}$ = 1.8 Hz, H-3), 2.90 (2 H, t, H-6'), 2.86 (1 H, dd, $J_{1eq,1ax}$ = 11.1 Hz, $J_{1eq,2}$ = 4.6 Hz, H-1eq), 2.64 – 2.55 (1 H, m, H-1'), 2.43 – 2.34 (1 H, m, H-1'), 2.28 – 2.23 (1 H, m, H-5), 2.00 (1 H, dd, $J_{1ax,2}$ = 10.7 Hz), 1.45 – 1.27 (13 H, m, H-5', H-2', C(C*H*3)3), 1.26 – 1.12 (4 H, m, H-3', H-4').δ_C (75 MHz, MeOD): 157.4 (NCO), 78.6 (*C*(CH_3)3), 76.1 (C-3), 71.0 (C-4), 67.8 (C-2), 64.0 (C-5), 61.2 (C-6), 56.3 (C-1), 52.8 (C-1'), 40.1 (C.6'), 29.8 (C-5'), 27.6 (3 C, C(*C*H_3)3), 27.1; 26.6 (C-3', C-4'), 23.8 (C-2'). *m/z*: 363.2 $(M + H)^+$, 366.43. $C_{17}H_{37}N_2O_6$ requires $M + H^+$ 366.567

***N*-[6-(benzyloxycarbonylamino)hexyl]-1,5-dideoxy-1,5-imino-D-galactitol (22):** To a solution of iminosugar **4** (370.5 mg, 2.27 mmol) and *N*-Cbz-6-aminohexyl-4-toluenesulfonate **21** [9] (3.05 g, 7.52 mmol) in DMF (16 mL), Na_2CO_3 (1.2 g, 11.32 mmol) was added and the reaction mixture stirred at 50 °C for 18 hours. After TLC showed complete conversion of the starting material, the solids were removed by filtration and the filtrate concentrated under reduced pressure. Silica gel column chromatography ($CHCl_3$/MeOH/NH_3 3:1:1 %, v/v) followed by treatment with basic ion exchange resin (Merck III, washed with water) gave compound **22** (688.9 mg, 1.7 mmol, 77 %) as a colourless syrup. $[\alpha]_D^{20}$ = -14.0 (*c* 1.2 in MeOH). δ_H (500 MHz, MeOD): 7.39 – 7.23 (5 H, m, Ph), 5.25 – 5-12 (2 H, m, NHCOOC*H*2Ph), 4.02-3.95 (1 H, m, H-4), 3.85 – 3.74 (3 H, m, H-2, H-6_a, H-6_b), 3.25 – 3.17 (1 H, dd, $J_{2,3}$ = 8.4 Hz, H-3), 3.10 (t, 2H, H-6'), 3.02 (1 H, bs, N*H*COOPh), 3.00 – 2.93 (1 H, m, H-1_{eq}), 2.74 – 2.64 (1 H, m, H-1'), 2.54 – 2.44 (1 H, m, H-1'), 2.39 – 2.32 (1 H, m, H-5), 2.10 (1 H, dd, $J_{1eq,1ax = 1ax,2}$ = 10.5 Hz, H-1ax), 1.58 – 1.43 (4 H, m, H-5', H-2'), 1.42 – 1.21 (4 H, m, H-3', H-4'). δ_C (125 MHz, MeOD): 157.8, (N*C*O), 137.2, 128.3, 127.8, 127.6 (6 C,

Ph), 76.1(C-3), 71.0 (C-4), 67.9 (C-2), 66.1 (OC*H*$_2$Ph), 64.0 (C-5), 61.2 (C-6), 56.9 (C-1), 52.8 (C-1’), 40.5 (C-6’), 29.7 (C-5’), 27.1, 26.5 (2 C, C-3’, C-4’), 23.8 (C-2’.) *m/z*: 397.29 $(M+H)^+$, $C_{20}H_{32}N_2O_6$ requires M + H^+ 397.548

References

1. Prade, H.; Mackenzie, L. F.; Withers, S. G. *Carbohydr. Res*. **1998**, *305*, 371-381.
2. Kempton, J. B.; Withers, S. G. *Biochemistry* **1992**, *31*, 9961-9969.
3. Leatherbarrow, R.J. GraFit Version 4.0, Erithacus Software, Staines, U.K., 1992.
4. Lai, E. C. K.; Morris, S. A.; Street, I. P.; Withers, S. G. *Bioorg. Med. Chem.* **1996**, *4*, 1929 – 1937.
5. Zhu, A.; Monaha, Z.; Zhang, Z.; Hurst, R.; Leng, L.; Goldstein, I. J. *Arch. Biochem. Biophys.* **1995**, *324*, 65 – 70-
6. Ly, H. D.; Howard, S.; Shum, K.; He, S.; Zhu, A.; Withers, S. G. *Carbohydr. Res.* **2000**, *329*, 539-547.
7. Hansen, J. B.; Buchardt, O. *J. Chem. Soc. Commun.* **1983**, *4*, 162 – 164.
8. Greimel, P.; Häusler, H.; Lundt, I.; Rupitz, K.; Stütz, A. E.; Tarling, C. A.; Withers, S. G.; Wrodnigg, T. M. *Bioorg. Med. Chem. Lett.* **2006**, *16*, 2067 – 2070.
9. Krivickas, S. J.; Tamanini E.; Todd, M. H.M; Watkinson, M, *J. Org. Chem.* **2007**, *72*, 8280 – 8289.
